# Single-cell Sequencing Traces Mitochondrial Transfers

**DOI:** 10.1093/gpbjnl/qzae092

**Published:** 2024-12-26

**Authors:** Mengying Wu, Weilin Pu, Zhenglong Gu

**Affiliations:** Fudan University, Shanghai 200438, China; Greater Bay Area Institute of Precision Medicine (Guangzhou), Guangzhou 510000, China; Greater Bay Area Institute of Precision Medicine (Guangzhou), Guangzhou 510000, China; Fudan University, Shanghai 200438, China; Greater Bay Area Institute of Precision Medicine (Guangzhou), Guangzhou 510000, China

## Mitochondrial transfer

Mitochondrial transfer, encompassing the transfer of whole mitochondria and mitochondrial DNA, manifests under specific biological conditions, and it holds potential to exert profound effects on various cellular functions, such as proliferation, differentiation, and metabolism. A pivotal discovery in 2006 illuminated that the active mitochondrial transfer from both adult stem cells and somatic cells could restore aerobic respiration in mammalian cells afflicted by nonfunctional mitochondria [1]. Moreover, adipocytes were found to transfer mitochondria to macrophages in white and brown adipose tissues to maintain metabolic homeostasis [2]. In addition, it has been elucidated that astrocytes can release functional mitochondria, which could be subsequently integrated into neurons following cerebral ischemia, contributing to the activation of endogenous neuroprotective and neurorecovery processes [3]. In the controlled environment of *in vitro* studies, it has been revealed that Rho0 cells, characterized by the absence of functional mitochondria, display the capacity to capture exogenous mitochondria sourced from supernatants or other cells. Remarkably, these captured mitochondria are harnessed by the Rho0 cells to restore deficiencies in aerobic respiration and bolster cell division processes [4]. These findings paint a vivid picture of mitochondrial transfer as a dynamic process that supports the exogenous replacement of damaged mitochondria, ultimately rescuing mitochondrial defects and the ensuing cell dysfunction.

In addition to automatic mitochondrial transfer, recent research shows that cancer cells can also hijack mitochondria from immune cells via physical nanotubes [5]. This phenomenon, characterized by the hijacking of mitochondria from T cells, holds significant implications for cancer immune escape and the unbridled proliferation of cancer cells. To visually confirm this phenomenon, field-emission scanning electron microscopy was employed to observe the moving mitochondria that had been labeled with fluorophores. These exciting discoveries provide a new insight for understanding tumor–immune interactions and cancer immune escape. However, the strategy of fluorophore-tagged mitochondria is impractical and cannot be applied under clinical conditions. Consequently, there is an imperative need to develop a pragmatic approach that enables the investigation of mitochondrial transfer among different cell types for human cancer research. Such an approach would deepen our knowledge of these intriguing interactions and hold promise toward clinical interventions with meaningful therapeutic implications.

## MERCI and its roles

Single-cell RNA sequencing (scRNA-seq) and single-cell assay for transposase-accessible chromatin sequencing (scATAC-seq) have significantly enriched our understanding of cell types as well as cell–cell interactions within the tumor microenvironment. Both scRNA-seq and scATAC-seq encapsulate a substantial volume of mitochondrial DNA and RNA reads. Notably, these single-cell sequencing datasets are readily accessible in clinical settings, serving as invaluable resources for investigating mitochondrial transfer between different cell types in patient samples.

In a recent study, the research teams led by Anlin Zhang and Bo Li have developed a single-cell deconvolution method called MERCI (mitochondrial-enabled reconstruction of cellular interactions) to quantitatively assess the mitochondrial transfer through the interrogation of scRNA-seq data [6]. The researchers first recapitulated the near-unidirectional T cell to cancer mitochondrial flow using fluorescence imaging with cocultured (CC) cancer cells and T cells, alongside monocultured (MC) cancer cells and primary CD8^+^ T cells. Subsequently, scRNA-seq profiling was performed on these three cohorts of cells, and a variant-calling pipeline (MERCI-mtSNP) was developed, scrutinizing single nucleotide variants (SNVs) within mitochondrial DNA molecules (mtSNVs). They revealed that the count of T cell-enriched mtSNVs was higher in CC cells than that in MC cells, while the count of T cell-depleted mtSNVs exhibited a marked decrease in CC cells. This result signifies the utility of scRNA-seq data for identifying genetic variants in the transferred mitochondrial genome.

Next, the researchers employed both the mtSNVs and the expression profiles of cancer and T cells, and applied the support vector regression (SVR) algorithm to identify mitochondrial receiver cells, achieving considerable accuracy. To accommodate the real-world samples with a scarcity of mitochondrial receiver cells, the researchers proposed a simple strategy involving the initial determination of mitochondrial receiver existence in a sample through permutation, followed by the discrimination of mitochondrial receiver cells using both DNA and RNA profiles. *In silico* analysis underscored the accuracy of MERCI in determining samples with over 7% true positive cells, which is deemed sufficiently robust for cancer samples. Given that a substantial portion of clinical studies relies on 10X Genomics scRNA-seq data, which frequently exhibit limited coverage of mtDNA, the researchers conducted additional coculture experiments by using the cutting-edge technology for mtDNA single-cell profiling (mtscATAC-seq) to acquire deep coverage on mtDNA. Their results demonstrated congruence between mtscATAC-seq and scRNA-seq data, affirming the applicability of MERCI in elucidating mitochondrial transfer events using scRNA-seq data.

Upon the application of MERCI to the scRNA-seq data derived from solid tumor samples, the researchers made noteworthy discoveries. They observed a pronounced up-regulation of genes associated with ATP synthesis, oxidative phosphorylation, cytoskeleton regulation, actin polymerization, and key constituents of the tumor necrosis factor-alpha (TNF-α) pathway in predicted mitochondrial transfer recipient cells. This observation suggested that cancer cells were enriched with mitochondrial-mediated energy production, while the TNF-α pathway emerged as a pivotal role in the initiation of nanotubes and the establishment of intercellular nanotube connectivity. In essence, these genes associated with nanotube formation could potentially serve as biomarkers for mitochondrial transfer.

To quantify mitochondrial transfer within tumor cells, the authors introduced a metric termed tumor mitochondrial transfer (TMT) score. This metric unveiled a significant correlation between mitochondrial transfer, cell cycle score, tumor hypoxia level, and diminished survival. Collectively, these findings imply that the acquisition of mitochondria by tumor cells promotes proliferation, ultimately resulting in reduced survival rates in various cancer patients.

## Conclusion and perspectives

Mitochondrial transfer is a common phenomenon observed among mammalian cells, notably exploited by cancer cells to hijack foreign mitochondria, enhancing their energy resources and inducing immune cell dysfunction. Zhang et al. [6] introduces a robust analytical framework, MERCI, for quantifying the degree of mitochondrial transfer by analyzing sc-RNA seq data — an unprecedented application in this context [7]. Notably, this method does not rely on mitochondrial tracking fluorescence or genetic manipulation, making it well-suited for clinical applications. Furthermore, this methodology can be further broadened to assess the mitochondrial transfer among various cell types. Consequently, the application of MERCI in assessing disease severity, prognostication, and identification of therapeutic targets spans a diverse spectrum of pathological conditions. It is known that the relevance of mitochondrial transfer is further underscored by its involvement in neurodegenerative diseases, where the acquisition of mitochondria by microglia plays a crucial role in disease progression. Thus, MERCI emerges as a viable means to quantify the extent of such transfers in the context of neurodegenerative diseases. To improve the clarity and transparency of MERCI, it is important to acknowledge its current limitations, including its limited sensitivity due to relatively low sequencing depths in standard 10X datasets and its inability to predict donor T cells. Users should be aware of these constraints when applying MERCI to clinical tasks such as prognosis or target identification. The collective implications of applying algorithms such as MERCI indicate promising prospects of its clinical utility in the future, hold the potential to confer tangible benefits to patients in terms of disease diagnosis and treatment, and unveil new dimensions of mitochondrial contributions to our cellular powerhouses.

## CRediT author statement

**Mengying Wu:** Writing – original draft. **Weilin Pu:** Writing – review & editing. **Zhenglong Gu:** Funding acquisition, Writing – review & editing. All authors have read and approved the final manuscript.
